# Anti-osteoporosis mechanism of resistance exercise in ovariectomized rats based on transcriptome analysis: a pilot study

**DOI:** 10.3389/fendo.2023.1162415

**Published:** 2023-08-17

**Authors:** Qing Wang, Heng Weng, Yue Xu, Hui Ye, Yongqi Liang, Lulu Wang, Yutong Zhang, Yujie Gao, Jiayi Wang, Yuchen Xu, Zhiling Sun, Guihua Xu

**Affiliations:** School of Nursing, Nanjing University of Chinese Medicine, Nanjing, China

**Keywords:** resistance exercise, transcriptome, ovariectomized rat model, postmenopausal osteoporosis, osteoclast

## Abstract

Postmenopausal osteoporosis is the main cause of fractures in women. Resistance exercise has a positive effect on bone mineral density in postmenopausal osteoporosis patients, but its mechanism is unclear. The purpose of this study was to explore the mechanism of resistance exercise in improving ovariectomized osteoporotic rats based on the transcriptome sequencing technique. Eighteen female Sprague-Dawley rats were randomly divided into the sham-operated group, the non-exercise group, and the resistance exercise group. The rat model of postmenopausal osteoporosis was established by bilateral ovariectomy. Ten weeks after the operation, the resistance exercise group received 2 weeks of adaptive training, and 12 weeks of resistance exercise began in the 13th week. The rats were trained 5 days per week, in 4 sets of 3 repetitions per day. After the intervention, all rats were sacrificed, and the body weight, bone mineral density, trabecular bone microarchitecture, and bone biomechanics were examined. At the same time, RNA-seq and enrichment analysis of gene ontology and Kyoto Encyclopedia of Genes and Genomes were performed on the left tibias, followed by Elisa and RT-qPCR verification. It had been found that resistance exercise can effectively counteract the weight gain of ovariectomized osteoporotic rats, and has a good effect on bone mineral density and trabecular bone microarchitecture. Enrichment analysis showed that regulation of gene expression and osteoclast differentiation is the most closely related biological process and signaling pathway shared by RE/Ovx and NE/Ovx groups. Our results revealed that resistance exercise can play a role in inhibiting osteoclast activation and preventing the enhancement of osteoclast bone resorption function in ovariectomized osteoporotic rats by inhibiting Fos/Fosb-regulated TRAP activation and relieving Calcr inhibition, which has important application value in preventing bone loss caused by estrogen deficiency.

## Introduction

Osteoporosis is a systemic metabolic skeletal disease characterized by low bone mass and microarchitecture destruction of bone tissue, predisposing to increased bone fragility and fractures, which has become a major public health problem worldwide. Women after menopause are more likely to suffer from osteoporosis than men (1). Postmenopausal osteoporosis (PMOP) is caused by an imbalance in bone formation and resorption due to estrogen deficiency. As its incidence rate continues to rise, it has brought a substantial economic and social burden (2, 3). Currently, drug therapy is the first-line treatment option, but suffers from side effects, poor compliance, and a lack of universal efficacy. Therefore, finding an alternative strategy that is simple, convenient, and free of side effects is crucial.

Exercise has been recommended as a non-pharmacological intervention in multinational osteoporosis guidelines, in which the common types of exercise are resistance exercise, aerobic exercise, vibration exercise, and so on (4–6). Among them, resistance exercise (RE) has been proven to have a prominent effect on improving bone mineral density (BMD) and reducing the risk of falls in osteoporosis patients (7, 8). Evidence-based guides have proposed that progressive resistance training has been recommended as an effective strategy to increase or maintain BMD for postmenopausal osteoporosis, which is effective in increasing muscle mass, size, and strength (9, 10). One meta-analysis has suggested that high-load resistance training can increase the BMD of the lumbar spine (11), however, the other meta-analysis presented that both high- and low-load resistance training have similar effects on femoral neck and lumbar spine BMD in aging people (12). The latest network meta-analysis has demonstrated RE is the most promising exercise type to increase the total hip BMD (13). However, due to the lack of relevant basic research, the mechanism of RE in improving postmenopausal osteoporosis is not clear, which limits the further optimization of the exercise regimen and application.

In recent years, multi-omics techniques have been increasingly applied in the exploratory studies of the pathogenesis and therapeutic molecular mechanism of OP. Transcriptomics, as a frequently used technique, is widely used to explore the associated mechanism of gene expression and transcription levels. It can rapidly and comprehensively analyze the changes in gene expression and transcription levels in specific tissue samples, obtain relevant transcript information, and easily and quickly screen out differential expression genes to help researchers focus on and lock in the pathogenesis and therapeutic mechanisms. Many researchers have applied transcriptomics to conduct exploratory studies on the pathogenesis of OP and the mechanism of aerobic exercise in OP (14–16), but no transcriptomics studies with resistance exercise in OP have been reported yet. Therefore, in order to explore the mechanism of resistance exercise in OP, this study used the ovariectomized rat model, the classical model for studying PMOP (17), to simulate the pathological changes of osteoporosis in postmenopausal women, and explored the specific mechanism of resistance exercise by transcriptome sequencing. The aim is to provide useful guidance for the application of resistance exercise in the clinical rehabilitation of postmenopausal osteoporosis.

## Materials and methods

### Experimental animals

Eighteen nine-week-old female Sprague-Dawley rats (weight 180-200g) were purchased from Shanghai SLAC Laboratory Animal Co.Ltd. (Shanghai, China). The rats were housed in the SPF Experimental Animal Center of Nanjing University of Chinese Medicine with a temperature of 20 to 26 °C and a 12/12 h light/dark cycle. The experimental animals were randomly divided into the sham-operated (Sham) group, the non-exercise (NE/Ovx) group, and the resistance exercise (RE/Ovx) group. All rats have free access to a standard laboratory diet and water. The experimental protocol was approved by the Ethics Committee for Animal Experiments of Nanjing University of Chinese Medicine (Ethics number: 202012A011).

### Ovariectomy protocol

After 1 week of adaptative feeding, the rats were anesthetized with 1% sodium pentobarbital through intraperitoneal injection (60 mg/kg body weight) and then received either a sham operation or bilateral ovariectomy. Six rats removed only a small piece of adipose tissue near the ovary in the Sham group, and the rats in the NE/Ovx and RE/Ovx groups removed the ovaries bilaterally. Erythromycin ointment was applied to the surgical wound of rats to prevent infection.

### Resistance exercise protocol

The climbing ladder for RE was purchased from Beijing Cinontech Co.Ltd., and the model is Lad-03. The ladder is 100 cm high, with each step spaced 2 cm apart and placed at a backward angle of 85°. The ladder is 14.5 cm wide and consists of stainless steel round tubes with a diameter of 3 mm. This ladder-climbing protocol was adapted from others who have published on resistance training in rats (18, 19). All rats in the RE/Ovx group received 2 weeks of adaptive training before the formal exercise. During the two-week acclimatization period, no weight bearing in the first week, and the training load in the second week was 50% of the rat’s body weight, meanwhile, the rats were trained 5 days per week, in 4 sets of 3 repetitions per day, and a weekend off, to familiarize the rats with the exercise regimen of ladder climbing.

**Figure 1:**
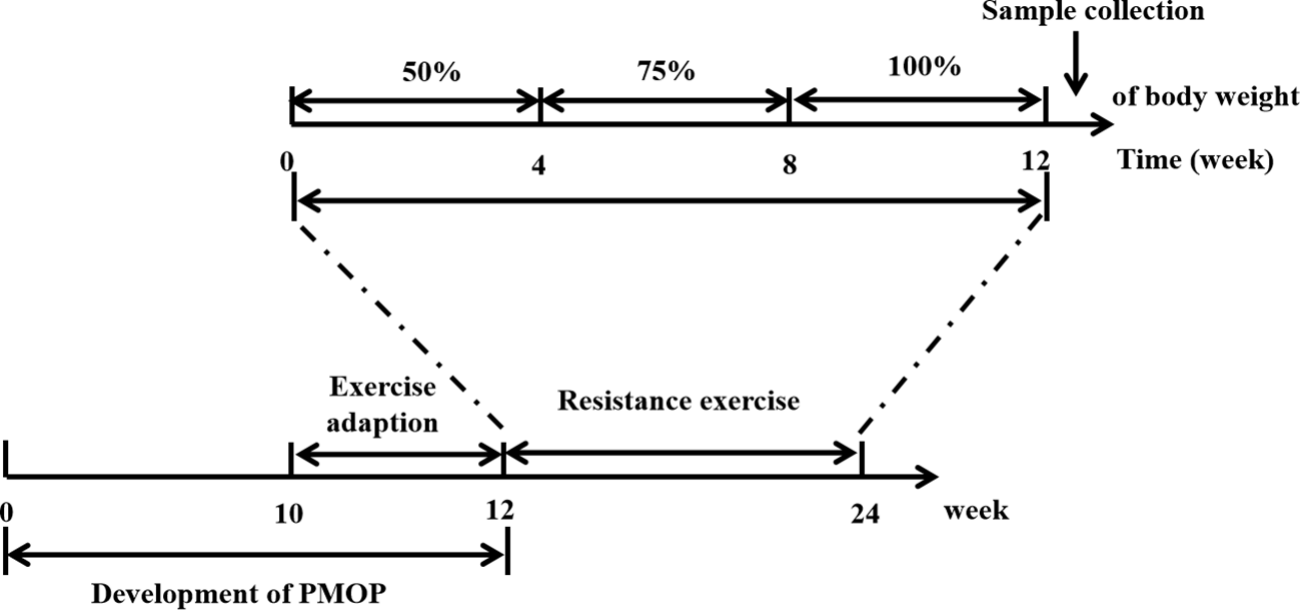
The schematic diagram of the study.

Formal training regimens began in the 13th week, and the rats in RE/Ovx group were intervened for 12 weeks. Each rat started at the bottom of the ladder and climbed 100 cm into the top storage chamber. It should be noted that all rats cannot be stimulated by electricity or heat to climb ladders. When the rats didn’t want to climb, the experimenter assisted them to start the first step on the ladder by lifting the tail, and then gently releasing the tail when they volunteered the second step was performed. All rats in the RE/Ovx group were encouraged by voice or soft touch during the climbing process until they voluntarily climbed to the top of the ladder. The rats were trained 5 days per week, in 4 sets of 3 repetitions per day, with 1 min rest at the top storage chamber of the ladder, and 2 min rest between sets for a total of 12 weeks. The weights and experimental steel balls were placed in a 50ml centrifuge tube that was attached to the bottom of the tail with exercise tape. The weight of weights and experimental steel balls was adjusted every Monday according to the weighed weight of the rats. The training load was set to crawl with 50% of the rat’s body weight for weeks 1 to 4, and 75% of the rat’s body weight for weeks 5 to 8, eventually reaching 100% for weeks 9 to 12. The schematic diagram of the study is shown in **Figure 1**.

### Sample collection

The body weights of rats in each group were measured and recorded before ovariectomy, after ovariectomy, and before weekly training during the exercise period. After 12 weeks of continuous exercise, blood samples were collected from the abdominal aorta under isoflurane anesthesia, and the rats were then sacrificed. The obtained plasma was stored at -80°C until measurement of bone turnover markers, the left femurs of rats were collected and immersed in saline for measuring bone biomechanics, and the right femurs were collected and stored at Paraformaldehyde until micro-computed tomography (micro-CT) analysis was performed. The left tibias were sent to Shanghai OE Biotech Co., Ltd. for transcriptome sequencing, and the right tibias were stored at -80°C for RNA extraction and real-time quantitative polymerase chain reaction (RT-qPCR) analysis.

### Micro-CT scanning analysis

BMD of cancellous bones (g·cm ^-3^) and trabecular bone microarchitecture were detected by the Micro-CT system (Hiscan XM Micro-CT, Suzhou Hiscan Information Technology Co., Ltd, Suzhou, China). The scan parameters were as follows: Voltage 80kV, current 100uA, single exposure time 50ms, scanning resolution 25um, and scanning angle interval 0.5 degrees. A region of interest (ROI) in the cancellous bone was selected for analysis, which was located near the metaphysis approximately 5 mm from the intercondylar notch. The images were reconstructed with Hiscan Reconstruct software and analyzed with Hiscan Analyzer software (Version 3.0, Suzhou Hiscan Information Technology Co., Ltd, Suzhou, China). Trabecular parameters: bone volume/total volume fraction (BV/TV), trabecular thickness (Tb.Th, mm), trabecular number (Tb.N, 1/mm), trabecular separation (Tb.Sp, mm) were measured.

### Biomechanical testing

The left femurs were removed from saline and restored to room temperature, dried with sterile gauze before testing, and the midpoint position was determined by length measurement along the long axis of the rat femoral shaft using vernier calipers. The bone was placed horizontally on the MTS Acumen3 biomechanical tester (MTS Systems Corporation, USA), the span of sample placement was adjusted to 1.5 cm, and pressure was applied vertically downward at a rate of 0.01 mm/s until the femur fractured and force data were recorded, from which the maximum load and stiffness were calculated.

### Transcriptome sequencing

The left tibias of three rats in each group (the Sham group, the NE/Ovx group, and the RE/Ovx group) were selected, and the skin, muscles, ligaments, and tendons were removed and sent to Shanghai OE Biotech Co., Ltd. for transcriptome sequencing. According to RNA-seq technical documents, total RNA was first extracted, and subsequently the Agilent 2100 Bioanalyzer (Agilent Technologies, Santa Clara, CA, USA) was used to evaluate the RNA integrity and construct libraries. These libraries were then sequenced on the Illumina sequencing platform (NovaSeq 6000).

### Differentially expressed genes and clustering analysis

Taking “osteoporosis” as the keyword, the DisGeNET database and OMIM database were searched and screened to obtain disease-related genes. The target results of the two databases were pooled and removed duplicates to obtain disease genes related to OP. The DESeq2 R (20) software was used for differential expression analysis of RNA-seq results, and differentially expressed genes (DEGs) with p-value < 0.05 and foldchange >1.2 or <0.83 were selected. The Venn diagrams were used to intersect the obtained disease-related genes. The differential mRNA enrichment of gene ontology (GO) and Kyoto Encyclopedia of Genes and Genomes (KEGG) were analyzed by hypergeometric distribution test.

### Elisa

The levels of tartrate-resistant acid phosphatase (TRAP) and beta-C-terminal telopeptide of type I collagen (β-CTX) in rat plasma samples were detected by ELISA kits (Shanghai MLBIO Biotechnology Co. Ltd., Shanghai, China) based on manufacturer’s instructions.

### RT-qPCR analysis

The tibias were ground with liquid nitrogen to isolate and extract total RNA, and the RNA was extracted using MolPure^®^ Bone RNA Kit (Yeasen Biotechnology Co., Ltd., Shanghai, China), reverse transcribed total RNA (1 μg) into cDNA, and subsequently subjected to quantitative PCR using GAPDH as the internal reference. qPCR reaction procedure: pre-denaturation at 95°C for 5min, denaturation at 95°C for 10s, and annealing at 60°C for the 30s, 40 cycles in total. The expression levels of the genes screened according to transcriptome sequencing were calculated by the 2 ^-ΔΔCt^ method. The primers are given in **Table 1**.

**Table 1:** The sequence of primers used for RT-Qpcr.

Gene	Forward	Reverse
Acp5	CTCAGCTGTCCTGGCTCAAA	ACTCAGCACATAGCCCACAC
Fos	GAGGGAGCTGACAGATACGC	TCCAGGGAGGTCACAGACAT
Fosb	TGGCCGAGGTGAGAGATTTG	GAAGGGCTAACAACGGGGAA
Calcr	GTGGGCCACTCCATGTCAAT	TGCAACTTATAGGATCCCGTCG
Stat1	GGAAGGGGCCATCACATTCA	CATCGGTTCTGGTGCTTCCT
GAPDH	GTTACCAGGGCTGCCTTCTC	GGGTTTCCCGTTGATGACC

### Statistical analysis

Statistical analysis was performed using GraphPad Prism 9.4.0 version (San Diego, USA), and data were expressed as mean ± standard deviation. Data normality was tested by the Shapiro-Wilk test. When comparing three groups, one-way ANOVA analysis was performed for normally distributed data, and a nonparametric test was used for non-normally distributed data. Two-way ANOVA was used in body weight among three groups.

## Results

### Effect of resistance exercise on body weight of ovariectomized rats

**Figure 2:**
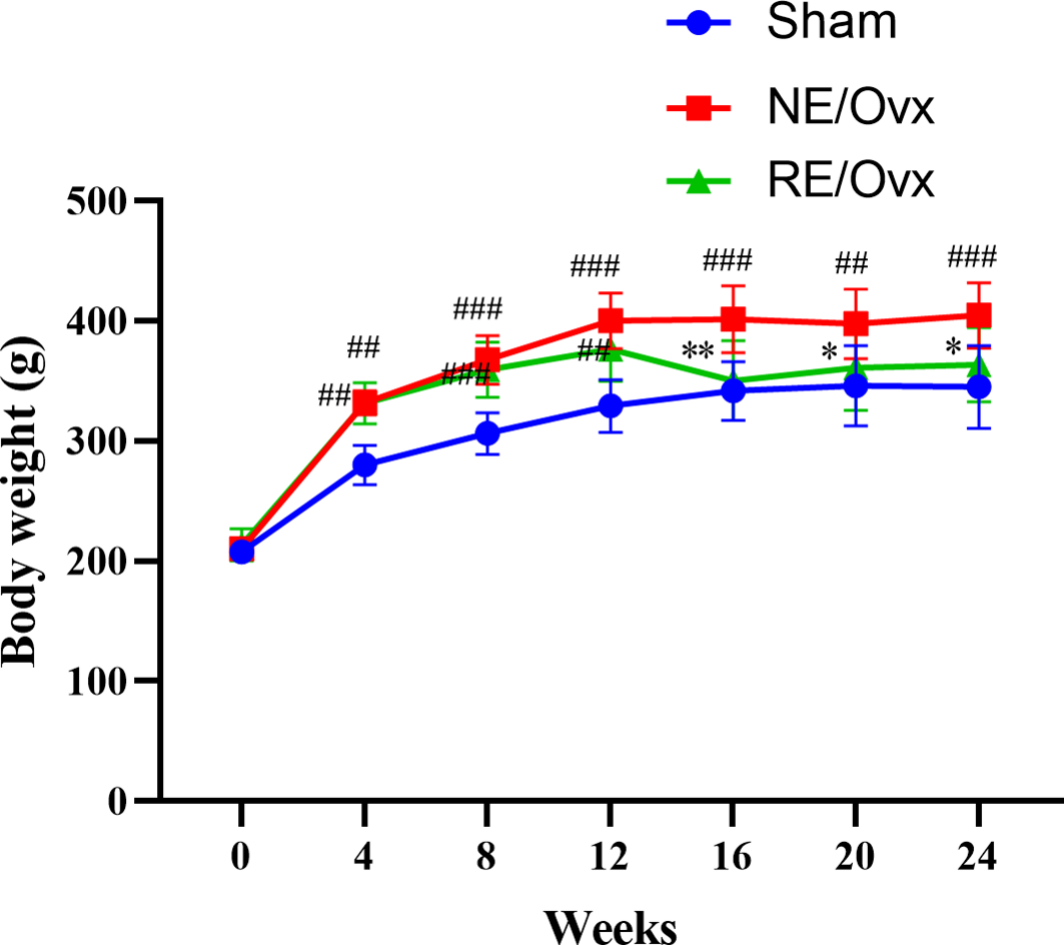
Effect of resistance exercise on body weight of Ovariectomized rat model. The data were expressed as mean ± SD (n=6). ^**^ *P* < 0.01, ^*^ *P* < 0.05 compared to the NE/Ovx group; ^###^ *P* < 0.001, ^##^ *P* < 0.01 compared to the Sham group.

Compared with the Sham group, the rats in the NE/Ovx group showed a significant increase in body weight in the 4th week after the operation (*P* < 0.01); in the 12th week after the operation, the body weight of the RE/Ovx group showed a downward trend due to adaptive exercise. During the formal exercise phase, the body weight of the RE/Ovx group decreased significantly compared with the NE/Ovx group, while there was no significant difference between the RE/Ovx group and the Sham group (as shown in **Figure 2**). It is suggested that there is a significant weight gain in ovariectomized rats, and resistance exercise with certain exercise intensity and duration can significantly improve this performance.

### Effects of resistance exercise on bone mineral density and trabecular bone microarchitecture in ovariectomized rats

**Figure 3:**
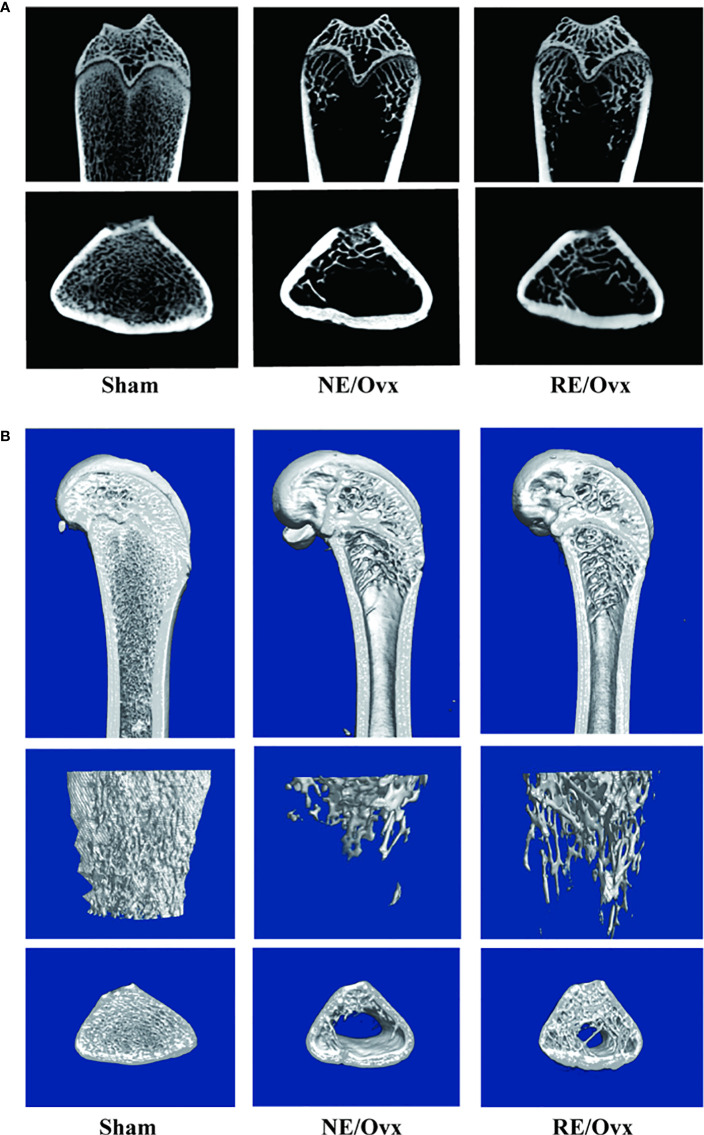
**(A)** Micro-CT 2D images in the trabecular architecture of distal femurs; **(B)** Micro-CT 3D images in the trabecular architecture of distal femurs (scale bar: 1mm).

**Figure 4:**
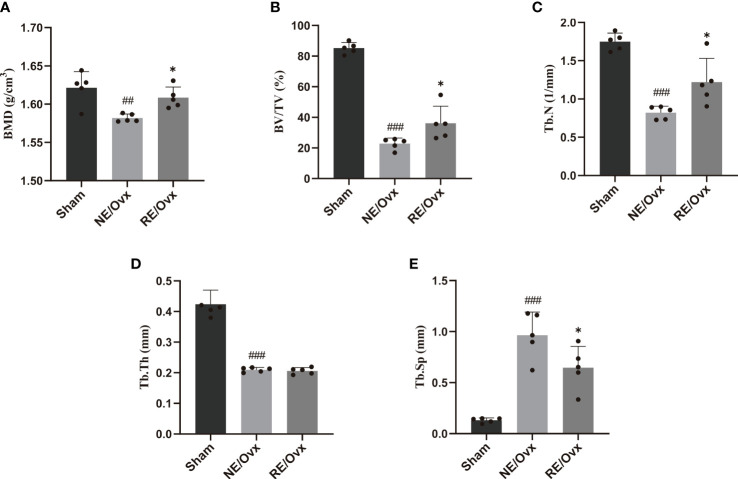
Quantitative results of **(A)** BMD and trabecular bone microarchitecture parameters: **(B)** BV/TV, **(C)** Tb.N, **(D)** Tb.Th, and **(E)** Tb.Sp. Graphs show mean ± SD (n=5). ^*^ *P* < 0.05 compared to the NE/Ovx group; ^###^P < 0.001, ^##^ *P* < 0.01 compared to the Sham group.

Micro-CT images showed that the number of trabecular was reduced in the NE/Ovx group compared with the Sham group, and its trabecular distribution was sparse (**Figure 3**). The results of micro-CT analysis showed that the BMD of cancellous bones, BV/TV, Tb.N, and Tb.Th in the NE/Ovx group was significantly reduced compared with the Sham group, while Tb.Sp was significantly increased compared with the Sham group (**Figure 4**). It is suggested that there is obvious osteoporosis in ovariectomized rats with good model formation and bone loss, and the trabecular was significantly decreased in terms of number, thickness, and density.

Meanwhile, micro-CT images showed that the number of trabecular was improved in the RE/Ovx group compared with the NE/Ovx group (**Figure 3**). And the results of micro-CT analysis also showed that compared with the NE/Ovx group, the BMD of cancellous bones, BV/TV, and Tb.N in the RE/Ovx group were significantly improved (*P <*0.05), Tb.Sp was significantly decreased (*P <*0.05) (**Figure 4**). It is suggested that resistance exercise can significantly improve osteoporosis and had the effect of counteracting the decrease in the number and density of trabecular bone and bone loss in ovariectomized rats.

### Effect of resistance exercise on bone biomechanical properties in ovariectomized rats

**Figure 5:**
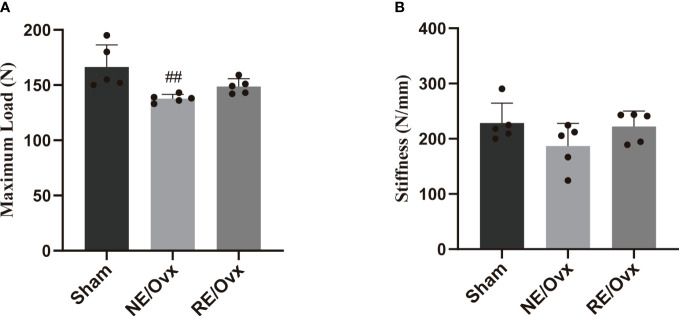
Effect of resistance exercise on bone biomechanical properties. **(A)** Maximum Load. **(B)** Stiffness. Graphs present mean ± SD (n=5). ^##^P < 0.01 compared to the Sham group.

The analysis of bone biomechanical properties revealed that the maximum load of rats in the NE/Ovx group was significantly lower than that of the Sham group (*P* < 0.01) (**Figure 5A**), while the stiffness of the NE/Ovx group rats also showed a certain degree of reduction (*P* > 0.05) (**Figure 5B**). Interestingly, there was no significant difference in stiffness among the Sham, NE/Ovx, and RE/Ovx groups (**Figure 5B**). It is suggested that there is no significant change in bone biomechanical properties in ovariectomized rats except for the maximum load.

Compared with the NE/Ovx group, the maximum load and stiffness of the RE/Ovx group showed a certain improvement trend (*P* > 0.05). It is suggested that resistance exercise has a more prominent effect on trabecular bone microarchitecture improvement, while there is also a certain trend of improvement in bone biomechanical properties, but the effect is not prominent (**Figure 5**).

### RNA-seq analysis of resistance exercise to improve osteoporosis in ovariectomized rats

Although resistance exercise has a protective effect on osteoporosis, its underlying mechanism is still unclear. Therefore, we explored its mechanism through transcriptomic analysis.

**Figure 6:**
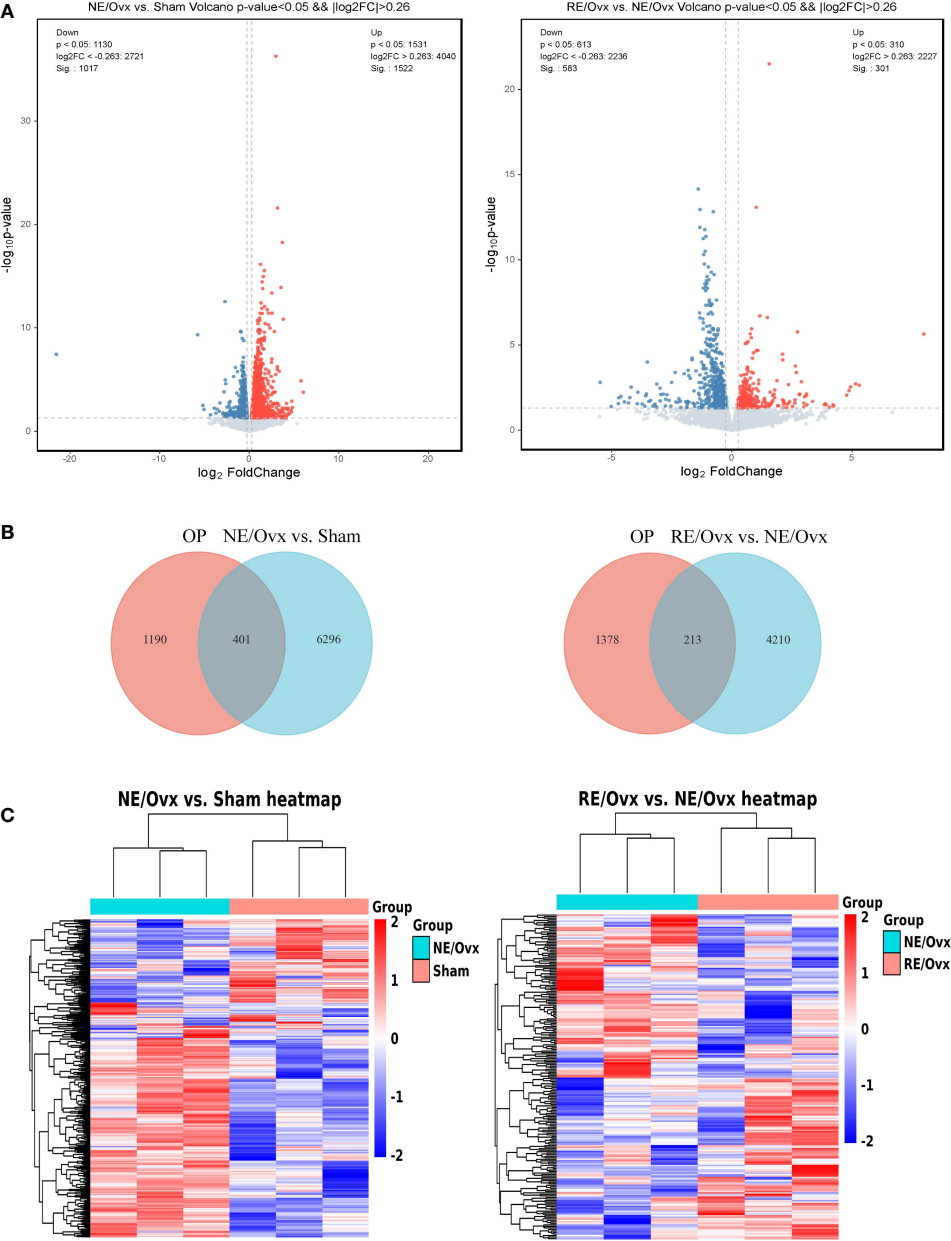
Analysis of DEGs. **(A)** Volcano plot of DEGs. **(B)** Venn diagram of DEGs between OP and NE/Ovx vs. Sham, OP and RE/Ovx vs. NE/Ovx. **(C)** Heatmap of 401 DEGs in NE/Ovx vs. Sham and 213 DEGs in RE/Ovx vs. NE/Ovx.

We found by RNA-seq that there were 6697 DEGs in the NE/Ovx group compared with the Sham group, of which 4040 were upregulated and 2657 were downregulated. There were 4423 DEGs in the RE/Ovx group compared with the NE/Ovx group, of which 2227 were upregulated and 2196 were downregulated (**Figure 6A**). To further explore the gene changes associated with osteoporosis among them, we retrieved 1591 OP-related disease genes through the DisGeNET database and OMIM database, and intersected these genes with the aforementioned 6697 DEGs in NE/Ovx vs. Sham and 4423 DEGs in RE/Ovx vs. NE/Ovx respectively, to obtain the target genes associated with the mechanism of resistance exercise to improve osteoporosis in ovariectomized rats explored in this study (**Figure 6B**).

Finally, 401 DEGs related to the mechanism of ovariectomized osteoporotic rats were obtained in the NE/Ovx group, including 284 upregulated and 117 downregulated genes, and 213 DEGs related to the improvement mechanism in the RE/Ovx group, of which 106 genes were upregulated and 107 downregulated (**Figure 6C**).

### Analysis of key regulatory pathways of resistance exercise to improve osteoporosis in ovariectomized rats

**Figure 7:**
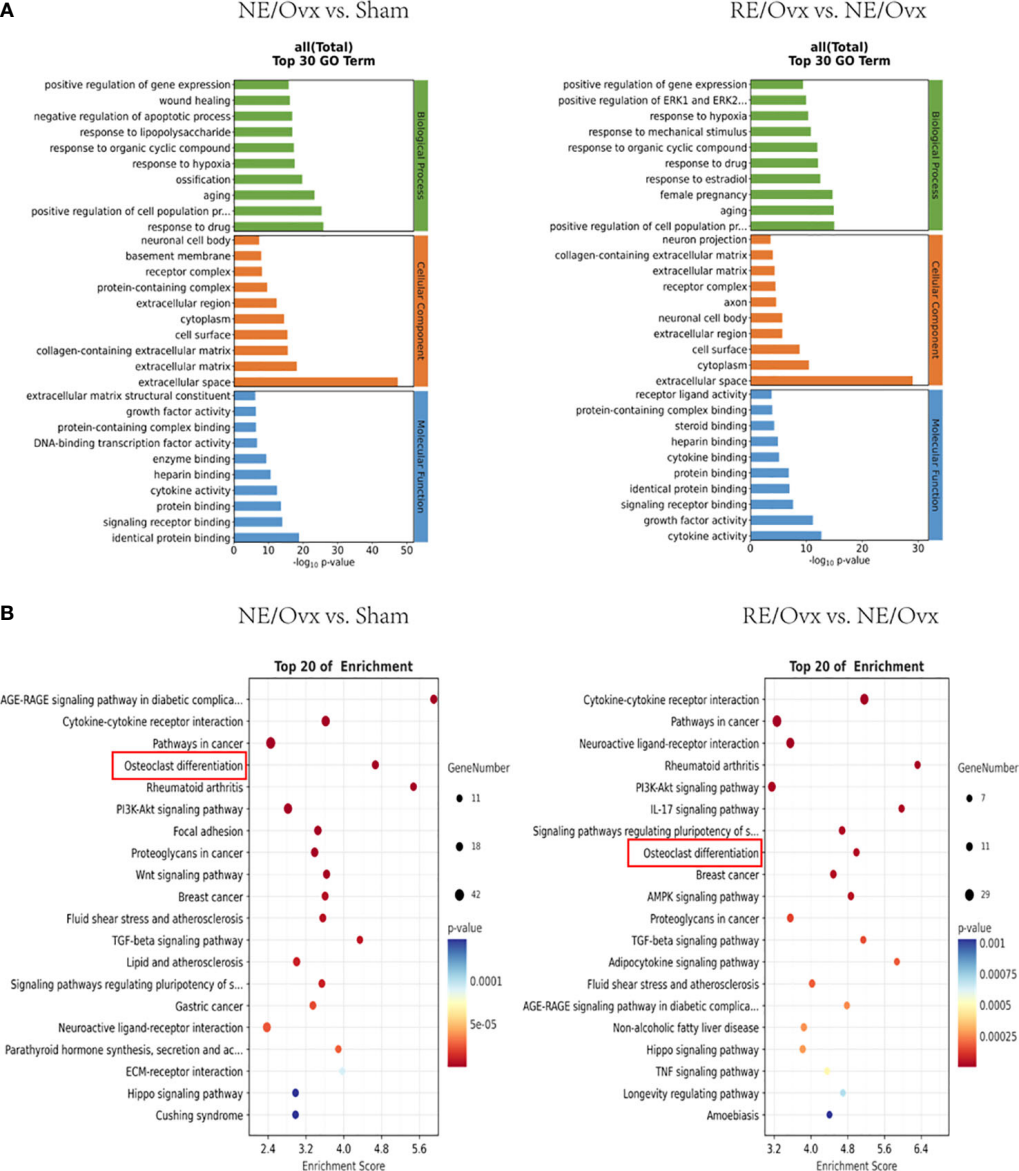
GO and KEGG enrichment analysis. **(A)** The top 30 GO terms of NE/Ovx vs. Sham and RE/Ovx vs. NE/Ovx. **(B)** The top 20 enrichment signaling pathways of NE/Ovx vs. Sham and RE/Ovx vs. NE/Ovx.

GO enrichment analysis of 401 DEGs related to the mechanism of ovariectomized osteoporosis in the NE/Ovx group showed that the biological process was mainly involved in aging, ossification, regulation of cell population proliferation, and regulation of gene expression. Cellular component mainly included the extracellular matrix, cell surface, cytoplasm, and extracellular region. Molecular function displayed enrichment in signaling receptor binding, cytokine activity, enzyme binding, and growth factor activity. GO enrichment analysis of 213 DEGs related to improving the mechanism of osteoporosis in the RE/Ovx group revealed that the biological process was mainly related to the regulation of cell population proliferation, aging, response to estradiol, response to mechanical stimulus, and regulation of gene expression. Cellular component mainly concerned extracellular space, cytoplasm, cell surface, and extracellular region. Molecular function exhibited enrichment in cytokine activity, growth factor activity, signaling receptor binding, cytokine binding, and receptor ligand activity (**Figure 7A**). From the above analysis, it can be found that biological process such as the regulation of gene expression is jointly involved in the mechanism of ovariectomized osteoporosis in the NE/Ovx group and the improvement of ovariectomized osteoporosis in the RE/Ovx group.

KEGG enrichment analysis of 401 DEGs related to the mechanism of ovariectomized osteoporosis in the NE/Ovx group showed that Cytokine-cytokine receptor interaction, osteoclast differentiation, PI3K-Akt signaling pathway, Focal adhesion, Wnt signaling pathway, TGF-beta signaling pathway, Neuroactive ligand-receptor interaction, Parathyroid hormone synthesis, secretion and action, ECM-receptor interaction, and Hippo signaling pathway were significantly enriched. Meanwhile, the KEGG enrichment analysis of 213 DEGs related to the improvement mechanism of ovariectomized osteoporosis in the RE/Ovx group showed that RE was mainly enriched with the Cytokine-cytokine receptor interaction, Neuroactive ligand-receptor interaction, PI3K-Akt signaling pathway, Osteoclast differentiation, AMPK signaling pathway, TGF-beta signaling pathway, Hippo signaling pathway, TNF signaling pathway (**Figure 7B**).

Among them, the osteoclast differentiation pathway not only participates in the mechanism of ovariectomized osteoporosis induced in the NE/Ovx group and the mechanism of improving ovariectomized osteoporosis in the RE/Ovx group, but is also highly enriched in the KEGG enrichment pathways of the above two groups, with an enrichment index greater than 4.5.

### Effect of resistance exercise on bone resorption function of osteoclasts

**Figure 8:**
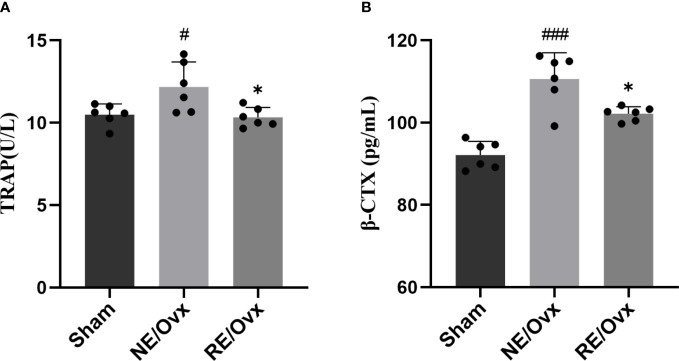
Effect of resistance exercise on bone resorption function of osteoclasts. **(A)** TRAP. **(B)** â-CTX. Graphs show mean ± SD (n=6). ^*^P < 0.05 compared to the NE/Ovx group; ^###^P < 0.001, ^#^P < 0.05 compared to the Sham group.

The above transcriptome analysis suggests that osteoclast differentiation may be the key mechanism of resistance exercise in improving ovariectomized osteoporosis, and TRAP and β-CTX are important indicators reflecting bone resorption of osteoclasts. In order to verify the above analysis results, the indicators were further detected. It was found that the levels of TRAP and β-CTX were significantly higher in the NE/Ovx group compared with the Sham group (*P* < 0.05), while they were significantly lower in the RE/Ovx group compared with the NE/Ovx group (*P* < 0.05) (**Figures 8A, B**). The above results suggested that osteoclast activation and bone resorption were significantly enhanced in ovariectomized rats, while resistance exercise could inhibit osteoclast activation in this process and prevent the enhancement of osteoclast bone resorption function.

### Regulatory effect of resistance exercise on key genes in the osteoclast differentiation pathway

**Figure 9:**
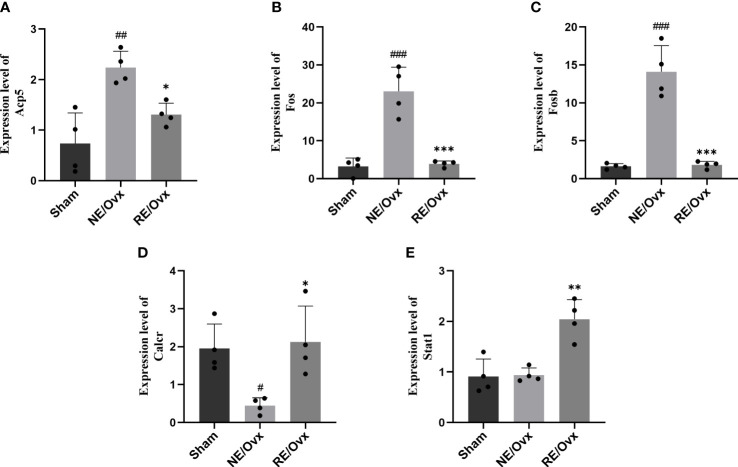
The mRNA expression levels of 5 DEGs. **(A)** Acp5. **(B)** Fos. **(C)** Fosb. **(D)** Calcr. **(E)** Stat1. Graphs represent mean ± SD (n=4). ^***^P < 0.001, ^**^P < 0.01, ^*^P < 0.05 compared to NE/Ovx group; ^###^P < 0.001, ^##^P < 0.01, ^#^P < 0.05 compared to the Sham group.

In order to further reveal how resistance exercise regulates osteoclast differentiation to improve bone resorption function, we analyzed the DEGs of the osteoclast differentiation pathway based on the above transcriptome analysis. Five DEGs with opposite expressions in the NE/Ovx group and the RE/Ovx group were screened and verified by RT-qPCR. It was found that Acp5, Fos, and Fosb genes were significantly elevated in the NE/Ovx group compared with the Sham group, while they were significantly downregulated in the RE/Ovx group compared to the NE/Ovx group (**Figures 9A–C**). Calcr was significantly reduced in the NE/Ovx group compared with the Sham group, while it was significantly upregulated in the RE/Ovx group compared with the NE/Ovx group (**Figure 9D**). The expression levels of the above four genes were consistent with the RNA-seq results. Meanwhile, although the expression level of Stat1 in the NE/Ovx group did not change significantly compared with the Sham group, it was significantly upregulated in the RE/Ovx group compared with the NE/Ovx group (**Figure 9E**). The above results indicate that Acp5, Fos, Fosb, and Calcr may be the key genes of the osteoclast differentiation pathway regulated by resistance exercise.

## Discussion

As an important means for the treatment of postmenopausal osteoporosis, exercise therapy has become an international consensus. However, due to the lack of relevant basic research, the mechanism of resistance exercise to improve postmenopausal osteoporosis is still unclear, which limits the further optimization of exercise protocol and its further application.

This study was conducted to investigate and find that resistance exercise can improve osteoporosis in ovariectomized rats, which is closely related to its effect on reducing bone loss by counteracting the decrease of trabecular number and density in ovariectomized rats.

On this basis, this study performed transcriptomic analysis on two comparative groups, Sham vs. NE/Ovx and NE/Ovx vs. RE/Ovx, to further reveal the potential mechanism of the improvement of bone metabolism in ovariectomized osteoporotic rats by resistance exercise. Since some of the DEGs identified by transcriptomics were not closely enough related to the disease in this study, we performed further intersection of the identified DEGs with known disease-related targets for subsequent analysis.

Through KEGG enrichment analysis of DEGs identified in Sham vs. NE/Ovx and RE/Ovx vs. NE/Ovx, it revealed that resistance exercise reversed several biological processes and signaling pathways affected by the pathological changes of osteoporosis, among which regulation of gene expression and osteoclast differentiation are the most closely related biological processes and signaling pathways shared by the RE/Ovx group and the NE/Ovx group with high enrichment index, suggesting that alteration of gene expression regulation of osteoclast differentiation pathway may be the core mechanism of resistance exercise to improve osteoporosis.

We know that osteoporosis is caused by the imbalance between osteoclast and osteoblast differentiation, and the activation of osteoclast differentiation and the enhancement of bone resorption are the important causes of OP. The above transcriptome analysis suggests that resistance exercise has a prominent reversal effect on this process. To confirm this effect, we further detected the indicators reflecting osteoclast bone resorption and found that resistance exercise does have the effect of inhibiting osteoclast activation and preventing the enhancement of osteoclast bone resorption.

Osteoclast activation involves multiple transcriptional regulatory pathways, such as the PI3K-Akt signaling pathway, NF-kappa B signaling pathway, MAPK signaling pathway, Calcium signaling pathway, JAK-STAT signaling pathway, et. al (21–24). Based on the DEGs of the osteoclast differentiation pathway in transcriptome analysis, we screened five DEGs with opposite expressions of the NE/Ovx group and the RE/Ovx group, belonging to the MAPK signaling pathway and JAK-STAT signaling pathway, in which Fos, Fosb, and Stat1 were also transcriptional regulatory factors. Subsequent validation by RT-qPCR revealed that Acp5, Fos, Fosb, and Calcr were significantly changed in both NE/Ovx and RE/Ovx groups, and their expression was completely consistent with the RNA-seq results.

Since TRAP produced by Acp5 transcription is a signature functional protein of osteoclast differentiation, and Fos and Fosb are important regulatory factors of Acp5 transcription (25), the inhibition of overactivation of functional proteins such as TRAP regulated by Fos/Fosb may be the core mechanism of resistance exercise to inhibit the osteoclast differentiation pathway and ameliorate ovariectomized osteoporosis. Meanwhile, CTR produced by Calcr transcription is an important receptor that receives calcitonin (CT) signal and enables CT to exert its inhibitory effect on osteoclast differentiation, so restoring Calcr transcription level and ensuring the feedback inhibitory effect of CT may also be an important mechanism for resistance exercise to inhibit osteoclast differentiation pathway and improve ovariectomized osteoporosis. In addition, although there was no significant difference in the NE/Ovx group, the level of Stat1 in the RE/Ovx group was significantly higher than that in the NE/Ovx group, suggesting that although Stat1 may not play an important role in ovariectomized osteoporosis, it may be involved in the above-mentioned mechanism of resistance exercise, which needs to be further explored in the following study.

In addition to the above mechanism for reversing the activation of osteoclast differentiation in ovariectomized osteoporotic rats, the transcriptome analysis results also suggested that resistance exercise had a potential regulatory effect on fluid shear stress and atherosclerosis, neuroactive ligand-receptor interaction, and TGF-beta signaling pathway. Among them, fluid shear stress and atherosclerosis is not only associated with atherosclerosis, but also an important pathway for mechanical stress to act on stress-receptor cells such as osteocytes, which can be activated by fluid shear stress (FSS) stimulation. Through membrane stress-sensitive ion channels, integrin/cytoskeleton complex, and gap junction, FSS physical signal was converted into the chemical signal (26, 27). Neuroactive ligand-receptor interaction is not only relevant to the nervous system, but also an important pathway for cellular communication in the bone microenvironment. For example, mesenchymal stem cells (MSCs) can be regulated by the sympathetic nervous system (SNS) and its transmitters, neuropeptides, and nerve growth factors (NGF) to show the activation of osteogenic differentiation, which makes it play a role in transmitting bone metabolism regulatory signals among cells in the bone microenvironment (28, 29). Besides, the action of TGF-β in bone metabolism is more prominent, and it can play a role in both osteoblasts and osteoclasts. On the one hand, TGF-β can promote osteogenic differentiation by acting on osteoblast progenitor cells; on the other hand, through secreting a large amount of TGF-β and binding to receptors on its own cell membrane to promote its own proliferation, differentiation, and extracellular matrix synthesis. Meanwhile, high concentration TGF-β can also inhibit osteoclast formation and differentiation, and even induce the programmed death of osteoclasts (30, 31). These results suggested that the mechanism of reversal of osteoclast differentiation and activation in ovariectomized osteoporotic rats by resistance exercise may not be isolated, but the result of coexistence with the above-mentioned mechanisms, and there may even be a chain change of osteoclast sensory stress, osteoblast differentiation activation, and osteoclast differentiation inhibition, all of which need to be further explored and confirmed by subsequent studies.

However, this study has some limitations. As the basic research on the mechanism of resistance exercise in osteoporosis is relatively few, the sample size of this study was mainly determined concerning related studies (32–35). However, at the end of this study, we conducted post-hoc power calculation and found that although the effect sizes were adequate for analyzing the improvement of BMD of cancellous bones, trabecular bone microarchitecture (BV/TV, Tb.Th, Tb.N, and Tb.Sp), and bone biomechanical properties indicator (maximum load) with this sample size (power value > 0.80), the effect size may be insufficient for bone biomechanical properties indicator (stiffness), so the sample size needs to be further increased in future to study the stiffness of bone biomechanical properties. Therefore, we defined this study as an exploratory study, and hope to further expand the sample size and conduct further research in the future as funding allows. In addition, we have chosen the current classical method of resistance exercise with ladder-climbing by referring to the relevant literature (18, 19). However, the reproducibility of this method to humans deserves further exploration. Indeed, in the upward stage, the rat needs to overcome its own resistance and the resistance of the weights to climb the ladder; while in the downward stage, the rats may climb the ladder mainly by overcoming the resistance of the weights, and the weights fall to produce the acceleration, which may also increase the resistance or additional impact force to some extent. Therefore, in order to better explore the resistance exercise, we will consider different ways of climbing up and down the ladder in the follow-up study.

In conclusion, our pilot study indicated that resistance exercise can play a role in inhibiting the activation of osteoclasts and preventing the enhancement of osteoclast bone resorption in ovariectomized osteoporotic rats by inhibiting Fos/Fosb-regulated TRAP activation and relieving Calcr inhibition, which has important application value in preventing bone loss caused by estrogen deficiency.

## Data availability statement

The raw data supporting the conclusions of this article will be made available by the authors, without undue reservation.

## Ethics statement

The animal study was reviewed and approved by the Ethics Committee for Animal Experiments of Nanjing University of Chinese Medicine (Ethics number: 202012A011).

## Author contributions

QW designed the experiments and wrote the draft of the manuscript. HW, YX, HY, YL, LW, YZ, YG, JW, and YCX participated in the animal exercise experiments. HW and QW analyzed the data. ZS and GX gave the research instructions. All authors contributed to the article and approved the submitted version.
